# ‘Decoy’ and ‘non-decoy’ functions of DcR3 promote malignant potential in human malignant fibrous histiocytoma cells

**DOI:** 10.3892/ijo.2013.1999

**Published:** 2013-06-28

**Authors:** MITSUNORI TODA, TERUYA KAWAMOTO, TAKESHI UEHA, KENTA KISHIMOTO, HITOMI HARA, NAOMASA FUKASE, YASUO ONISHI, RISA HARADA, MASAYA MINODA, MASAHIRO KUROSAKA, TOSHIHIRO AKISUE

**Affiliations:** 1 Department of Orthopaedic Surgery, Kobe University Graduate School of Medicine, Chuo-ku, Kobe 650-0017, Japan; 2 NeoChemir Inc., Sannomiya Chuo-building 4F, Chuo-ku, Kobe 651-0087, Japan

**Keywords:** DcR3, apoptosis, migration, invasion, matrix metalloproteinase

## Abstract

Decoy receptor 3 (DcR3) is a soluble secreted protein that belongs to the tumor necrosis factor receptor (TNFR) superfamily. DcR3 inhibits the Fas ligand (FasL)/Fas apoptotic pathway by binding to FasL, competitively with Fas receptor. Previous studies have reported that overexpression of DcR3 has been detected in various human malignancies and that DcR3 functions as a ‘decoy’ for FasL to inhibit FasL-induced apoptosis. In addition, recent studies have revealed that DcR3 has ‘non-decoy’ functions to promote tumor cell migration and invasion, suggesting that DcR3 may play important roles in tumor progression by decoy and non-decoy functions. We have previously reported that overexpression of DcR3 was observed in human malignant fibrous histiocytoma (MFH), however, the roles of DcR3 in MFH have not been studied. In the present study, to elucidate the roles of DcR3 in tumor progression of MFH, we examined the effects of DcR3 inhibition on cell apoptosis, migration and invasion in human MFH cells. siRNA knockdown of DcR3 enhanced the FasL-induced apoptotic activity and significantly decreased cell migration and invasion with a decrease in the activation of phosphatidylinositol 3 kinase (PI3K)/Akt and matrix metalloproteinase (MMP)-2. The findings in this study strongly suggest that DcR3 plays important roles in tumor progression of human MFH by decoy as well as non-decoy functions and that DcR3 may serve as a potent therapeutic target for human MFH.

## Introduction

Malignant fibrous histiocytoma (MFH), which has recently been classified as undifferentiated pleomorphic sarcoma (UPS), is the most common high-grade soft tissue sarcoma that occurs in late adult life (1,2). Advances in the treatment of MFH have led to multidisciplinary treatment, including surgery, chemotherapy and radiation therapy, resulting in great improvements in the quality of life of patients with the disease, however, the chemotherapy and radiation therapy strategies for MFH are not as effective as those for other malignancies. After adequate local treatment and adjuvant therapy, ~50% of patients invariably relapse with local recurrence and distant metastasis, hence the prognosis of patients with MFH is poor (1,3,4). Therefore, it is necessary to elucidate the factors contributing to tumor progression and metastasis in MFH and to establish more effective therapeutic strategies against MFH.

Decoy receptor 3 (DcR3) is a newly identified member of the tumor necrosis factor receptor (TNFR) superfamily. DcR3 lacks a transmembrane domain and is thought to be a soluble secreted protein. DcR3 is known to act as a decoy receptor for Fas ligand (FasL) (5), LIGHT (6) and TL1A (7). Among these, FasL, which is produced by activated T cells and natural killer cells, is the most important regulator of cellular apoptosis through the death receptor pathway. DcR3 competes with Fas receptor for binding to FasL and inhibits the FasL/Fas apoptotic pathway. Fas receptor has an intracellular death domain that triggers the extrinsic apoptotic signaling pathway by activating caspase-8, which can induce apoptosis through the activation of caspase-3, -6 and -7 and poly (ADP-ribose) polymerase (PARP). The FasL/Fas apoptotic pathway is a central regulator of apoptosis in mammals to eliminate malignant tumor cells and resistance to this apoptotic pathway is thought to be one of the hallmarks of malignant tumors (8,9). Therefore, DcR3 has been thought to contribute to tumor progression by inhibiting FasL-induced apoptosis of tumor cells.

Recent studies have demonstrated that DcR3 also functions as an effector molecule independently of the FasL/Fas apoptotic pathway. DcR3 overexpression promotes migration and invasion of nasopharyngeal carcinoma cells (10). In breast cancer cells, DcR3 suppression decreases both migration and invasion (11). Moreover, several studies have revealed that DcR3 promotes migration of HUVECs with an increased expression of matrix metalloproteinase (MMP)-2 (12) and that DcR3 can activate various signaling kinases such as Akt, extracellular signal-regulated kinase 1/2 (ERK1/2), c-Jun N-terminal kinase (JNK) and p38 mitogen-activated protein kinase (p38) (13–17). These reports strongly indicate that DcR3, in addition to its role as a decoy receptor for FasL, may function as a modulator of malignant progression in cancer cells.

Overexpression of DcR3 has been reported in various malignancies such as lung and colon cancers (5), EBV or HTLV-1 associated lymphomas (18), malignant gliomas (19) and pancreatic adenocarcinomas (20). Furthermore, we have previously reported that overexpression of DcR3 was observed in bone and soft tissue sarcomas (21). Several studies have also revealed that DcR3 overexpression is associated with distant metastasis and overall survival in human cancers (22–27). In the development of metastasis, MMPs are thought to play important roles in tumor cells by degrading the extracellular matrix (ECM) (28,29). The increased expression of certain MMPs correlates with tumor expansion, invasiveness and poor prognosis of patients with malignant tumors (30). Among the MMP subtypes, activation of MMP-2 has been observed in MFH (31,32), therefore, MMP-2 is thought to contribute to the metastatic potential of human MFH.

Based on previous studies, DcR3 may contribute to tumor progression by not only neutralizing FasL-induced apoptosis but also by promoting tumor migration and invasion, which are important for the development of metastasis. We hypothesized that DcR3 may play important roles in tumor progression and metastatic potential in human MFH through the inhibition of the apoptotic pathway and the signaling pathway that is related to migration and invasion. In the present study, we evaluated the effects of DcR3 inhibition on cell apoptosis, migration and invasion using human MFH cell lines to elucidate the roles of DcR3 in human MFH.

## Materials and methods

### Human MFH cell lines

Two human MFH cell lines, TNMY1 and Nara-H, were used in this study. TNMY1 was previously established in our laboratory (33) and Nara-H was obtained from ScienStuff Co. (Nara, Japan) (34). Cells were grown in culture medium consisting of Dulbecco’s modified Eagle’s medium (DMEM; Sigma-Aldrich Co., St. Louis, MO, USA) supplemented with 10% fetal bovine serum (FBS; Sigma-Aldrich) and 100 U/ml penicillin/streptomycin solution (Sigma-Aldrich). Cell lines were routinely maintained at 37°C in a humidified 5% CO_2_ atmosphere. For all experiments, we used the DMEM containing 10% FBS without the antibiotic solution.

### Transfection of small interfering RNA (siRNA)

To evaluate the effect of DcR3 knockdown, we transfected MFH cell lines with DcR3-specific small interfering RNA (siRNA). Briefly, 1 day before transfection, cells were seeded in 6-well culture plates in growth medium. Then, cells were transfected with 60 nmol of either a specific siRNA against human DcR3 (DcR3-si) (Invitrogen, Carlsbad, CA, USA) or a negative control siRNA (Ctrl-si) (Invitrogen) using Lipofectamine 2000 transfection reagent according to the manufacturer’s protocol (Invitrogen).

### Recombinant Fc, FasL and PI3K inhibitor treatment

After siRNA transfection, DcR3-si transfected cells were incubated in medium with 0 (DcR3-si cells) or 3 μg/ml of recombinant DcR3-Fc (DcR3-si+DcR3-Fc cells; R&D Systems, Minneapolis, MN, USA), or 3 μg/ml of recombinant IgG-Fc (DcR3-si+IgG-Fc cells, as a control; R&D Systems) for 24 h. Ctrl-si transfected cells were incubated without any recombinant Fc proteins (Ctrl-si cells).

To induce apoptosis, cells were treated with FasL (100 ng/ml; Peprotech, Rocky Hill, NJ, USA) for 6 h after siRNA transfection and Fc treatment.

To evaluate kinase activities, cells were pretreated with 0 or 20 μM of LY294002 [a phosphatidylinositol 3 kinase (PI3K) inhibitor; Cell Signaling Technology, Danvers, MA, USA] in DMSO (Wako, Osaka, Japan) for 2 h followed by a 1-h treatment with DcR3-Fc or IgG-Fc.

### Quantitative real-time PCR

We isolated total RNAs from cell lines using an RNeasy Mini kit according to the manufacturer’s protocol (Qiagen, Valencia, CA, USA) and first strand cDNAs were transcribed. Quantitative real-time PCR (qRT-PCR) was performed in a 20-μl reaction mixture using the Power SYBR Green Master Mix reagent (Applied Biosystems, Foster City, CA, USA) on an ABI PRISM 7500 sequence detection system (Applied Biosystems). The PCR conditions were as follows: 1 cycle at 95°C for 10 min followed by 40 cycles at 95°C for 15 sec and 60°C for 1 min. Primers for human *DcR3*, *MMP-2* and human *β-actin* (control) were synthesized by Hokkaido System Science (Hokkaido, Japan). Primer used were: *DcR*3: 5′-TCAATGTGCCAGGCTCTTC-3′ and 5′-AGCCACAAAG TCGATGACG-3′; *MMP-2*: 5′-ACAGCAGGTCTCAGCC TCAT-3′ and 5′-TGCCTCTGGACAACACAGAC-3′; *β-actin*: 5′-GATGAGATTGGCATGGCTTT-3′ and 5′-CACCTTCA CCGTTCCAGTTT-3′. The values were normalized with those for β-actin and relative expression was analyzed using the ΔΔCt method.

### Immunoblot analysis

Lysates were extracted from cells using a whole cell lysis buffer (Mammalian Protein Extraction Reagent, Thermo Scientific, Rockford, IL, USA) supplemented with a protease and phosphatase inhibitor mix (Roche Applied Science, Indianapolis, IN, USA). The protein content of lysates was then quantified using BCA Protein Assay reagent (Bio-Rad, Richmond, CA, USA). Samples containing equal amounts of protein were electrophoresed through 12% polyacrylamide gels and transferred onto PVDF membranes. After blocking membranes were incubated overnight at 4°C with the following antibodies in CanGet Signal Solution 1 (Toyobo Co., Ltd., Osaka, Japan): anti-human DcR3 (1:1,000), anti-human Fas (1:1,000), anti-human PARP (1:1,000), anti-human cleaved PARP (1:1,000), anti-human caspase-3 (1:1,000), anti-human cleaved caspase-3 (1:500), anti-human MMP-2 (1:1,000), anti-human Akt (1:2,000), anti-human phosho-Akt (p-Akt; 1:1,000), anti-human ERK1/2 (1:2,000), anti-human phospho-ERK1/2 (p-ERK1/2; 1:1,500), anti-human JNK (1:1,000), anti-human phospho-JNK (p-JNK; 1:1,000), anti-human p38 (1:1,000), anti-human phospho-p38 (p-p38; 1:1,000). All antibodies were purchased from Cell Signaling Technology. Following washes, membranes were incubated with the appropriate secondary antibody conjugated to horseradish peroxidase and were exposed with ECL Plus western blot detection system reagent (GE Healthcare Biosciences, Piscataway, NJ, USA). Antibody binding was detected by Chemilumino analyzer LAS-3000 mini (Fujifilm, Tokyo, Japan). Membranes were reprobed with anti-human α-tubulin antibody (Sigma-Aldrich) to confirm equal protein loading.

### Cell proliferation assays

To evaluate the involvement of DcR3 in MFH cell proliferation, we performed WST-8 cell proliferation assay using Cell Counting Kit-8 (CCK-8; Dojindo Inc., Kumamoto, Japan). Cells were seeded in 96-well culture plates at a density of 5×10^3^ cells/well in 100 μl culture medium. After siRNA transfection and Fc treatment, cells were treated with or without FasL to induce apoptosis. At the indicated incubation times (0, 24 and 48 h), 10 μl of the CCK-8 solution was added into each well and incubated for 1 h. Optical density was measured at a wavelength of 450 nm using a Model 680 Microplate Reader (Bio-Rad, Hercules, CA, USA). The relative number of viable cells in each well was calculated.

### Cell migration assays

To evaluate the effect of DcR3 on MFH cell migration, we performed *in vitro* scratch wound healing assays as previously described (35). Cells in 6-well culture plates were transfected with siRNA and treated with recombinant Fc and then incubated to form a confluent monolayer. A denuded area was created by scraping with a sterile 200-μl pipette tip and each well was washed three times with PBS to remove floating cells. Scratch wounds were inspected with an inverted microscope (Zeiss, Oberkochen, Germany) and captured by Motic Images Plus 2.2S (Shimadzu, Kyoto, Japan) after 0, 12 and 24 h of wounding. The distance between the opposing edges of the wound was measured at three points and averaged on each image.

### Cell invasion assays

The effect of DcR3 on cell invasion was assessed by Transwell chamber invasion assays, as previously described (36). After siRNA transfection and Fc treatment, 5×10^4^ cells were placed in the upper wells of 24-well Transwell chambers (BioCoat Matrigel Invasion Chamber, BD Biosciences, Bedford, MA, USA) and the lower wells were filled with complete growth medium. The chambers were incubated for 30 h to allow cells to invade from the upper wells towards the lower wells. After incubation, non-invading cells on the upper surface of membranes were removed by scrubbing and invading cells on the lower surface of the membranes were fixed, inspected with a microscope and imaged. The number of invading cells was counted in three random fields.

### Gelatin zymography

To evaluate the enzyme activity of MMP-2, we performed gelatin zymography as previously described (37). The cell culture supernatant in each well was collected and concentrated through an Amicon Ultra-4 10,000 MWCO Centrifugal Filter Device (Millipore, Billerica, MA, USA) and samples were electrophoresed through 10% gelatin gels (Invitrogen). After electrophoresis, the gels were washed with renaturing buffer (Invitrogen) for 30 min, followed by incubation with developing buffer (Invitrogen) overnight at 37°C. The gels were stained with Coomassie Brilliant Blue R-250 Staining Solution (Bio-Rad) and clear bands of MMP-2 were visible against the dark blue background.

### Statistical analysis

Each experiment was performed independently at least three times and data are presented as the mean ± standard deviation (SD). The statistical significance of the differences among means was evaluated by ANOVA with post hoc test. Results were considered significant at P<0.05.

## Results

### DcR3 knockdown enhanced FasL-induced apoptosis in human MFH cells

**Figure 1:**
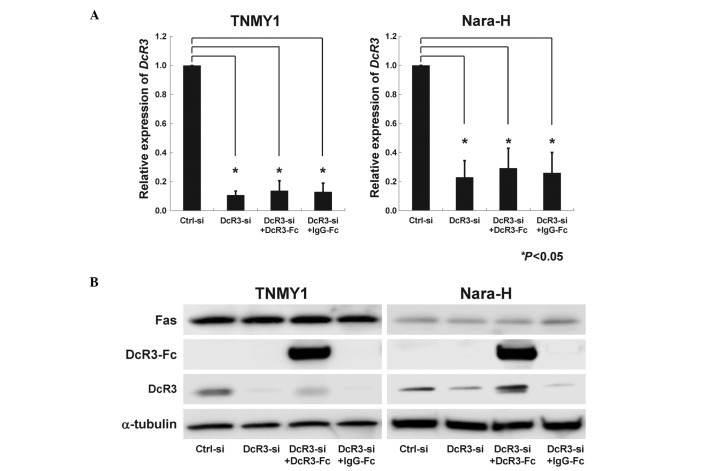
siRNA knockdown of DcR3 and DcR3-Fc treatment in MFH cell lines. TNMY1 and Nara-H cells were transfected with either a specific siRNA against DcR3 (DcR3-si) or a negative control siRNA (Ctrl-si). Then, DcR3-si transfected cells were treated with PBS (DcR3-si), recombinant DcR3-Fc (DcR3-si+DcR3-Fc), or recombinant IgG-Fc (DcR3-si+IgG-Fc) for 24 h. (A) *DcR3* mRNA expression was analyzed by qRT-PCR (^*^P<0.05). (B) Protein expressions of DcR3, DcR3-Fc and Fas were evaluated by immunoblot analysis.

We performed transfection of DcR3-siRNA and DcR3-Fc treatment to evaluate the effects of DcR3 in MFH cells. In both MFH cell lines, DcR3-siRNA transfection strongly suppressed DcR3 mRNA (^*^P<0.05, Fig. 1A) and protein expression levels (Fig. 1B). DcR3-Fc treatment slightly, but not significantly, increased DcR3 expression, while IgG-Fc treatment did not affect DcR3 expression (Fig. 1B). Fas expression was not affected by DcR3-siRNA transfection or DcR3-Fc treatment (Fig. 1B).

**Figure 2:**
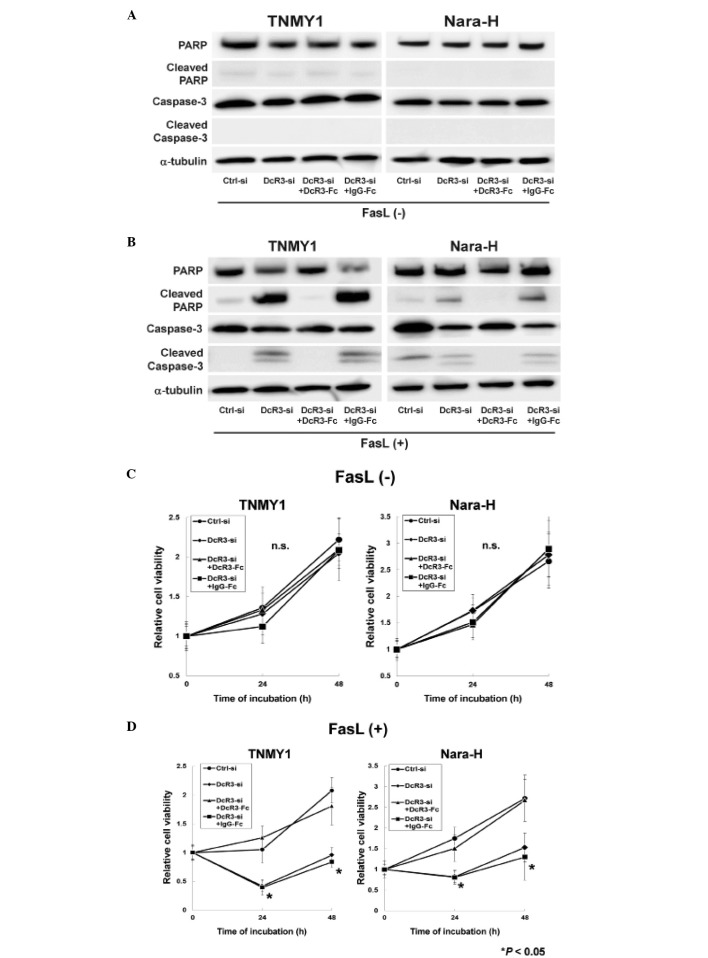
Effects of DcR3 knockdown with or without FasL treatment on apoptotic activity and cell proliferation in human MFH cells. After siRNA transfection and recombinant Fc treatment, cells were incubated for 6 h with or without FasL (100 ng/ml) to induce apoptosis. (A and B) Immunoblot analysis of caspase-3, PARP and their cleaved forms. (C and D) Cell proliferation assays after 24 and 48 h of incubation (^*^P<0.05).

Immunoblot analysis revealed that DcR3 knockdown or DcR3-Fc treatment without FasL treatment did not affect the expressions of caspase-3, PARP and their cleaved forms in either cell line (Fig. 2A). FasL treatment strongly induced the cleavage of caspase-3 and PARP in DcR3-si and DcR3-si+IgG-Fc cells, while the cleaved forms of capase-3 and PARP were barely detected in Ctrl-si cells (Fig. 2B). The increased expressions of cleaved capase-3 and cleaved PARP were suppressed by DcR3-Fc treatment in both MFH cell lines (DcR3-si+DcR3-Fc cells; Fig. 2B).

A previous report demonstrated that DcR3 knockdown with FasL treatment significantly suppressed cell proliferation in human pancreatic adenocarcinoma cells (38), therefore, we evaluated the effect of DcR3 inhibition with FasL treatment on MFH cell proliferation. siRNA transfection and recombinant Fc treatment without FasL treatment did not affect cell proliferation in either cell line (Fig. 2C), whereas, cell proliferation was significantly decreased in DcR3-si and DcR3-si+IgG-Fc cells with FasL treatment (^*^P<0.05, Fig. 2D). Moreover, DcR3-Fc treatment increased cell proliferation in DcR3-si cells to the same levels as in Ctrl-si cells (DcR3-si+DcR3-Fc cells; Fig. 2D).

### DcR3 knockdown significantly suppressed MFH cell migration and invasion

**Figure 3:**
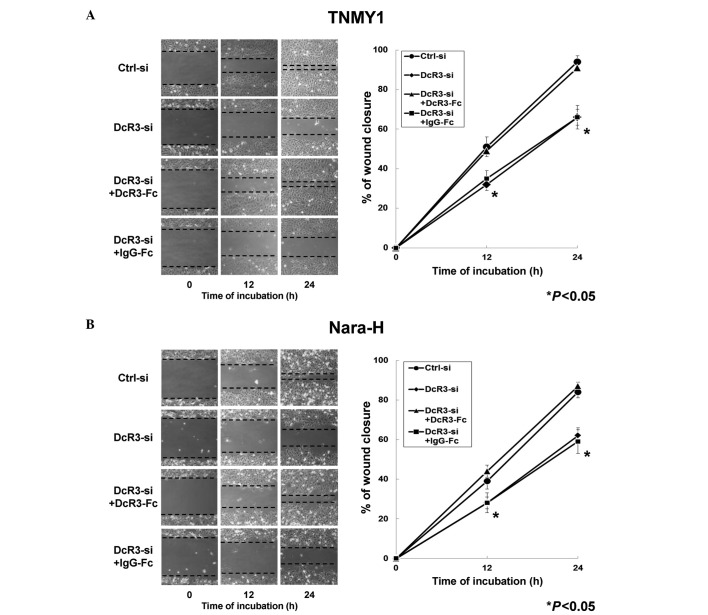
Effect of DcR3 on MFH cell migration. *In vitro* scratch wound healing assays of siRNA transfected MFH cell lines [TNMY1 (A) and Nara-H (B)] treated with recombinant Fc was used to evaluate cell migration. Dotted lines indicate the margins of migrating cells. The average wound distance of cell migration into the wound surface was determined under an inverted microscope 0, 12 and 24 h after wounding (^*^P<0.05).

We evaluated the effects of DcR3 on MFH cell migration and invasion to investigate the non-decoy functions of DcR3 in human MFH. In the *in vitro* scratch wound healing assays, DcR3 knockdown significantly decreased cell migration in both MFH cell lines at 12 and 24 h after wounding and DcR3-Fc treatment significantly restored the migration ability compared with control cells, respectively (^*^P<0.05, Fig. 3). Significant changes in cell migration were not observed in either MFH cell line with IgG-Fc treatment (Fig. 3).

**Figure 4:**
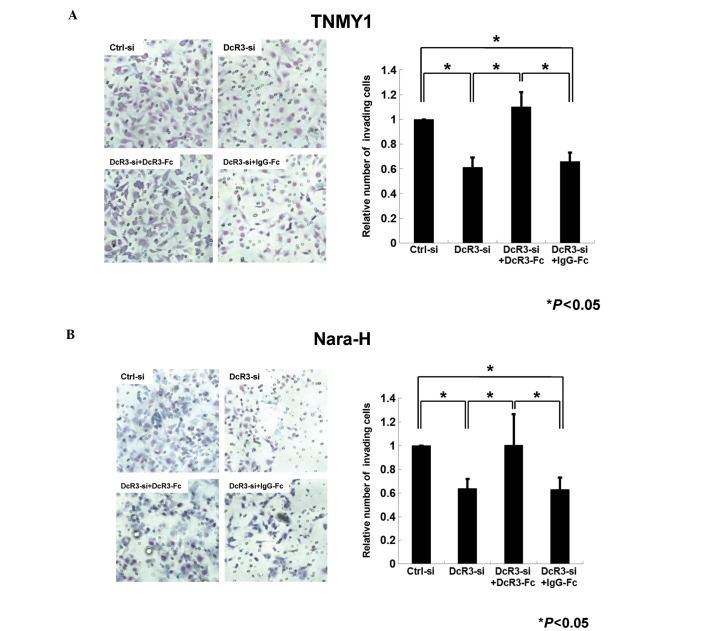
Effect of DcR3 on MFH cell invasion was assessed by transwell chamber invasion assays [TNMY1 (A) and Nara-H (B)]. After siRNA transfection and recombinant Fc treatment, cells were placed in the upper wells of 24-well transwell chambers. The chambers were incubated for 30 h and invading cells on the lower surface of the membranes were inspected with a microscope and counted in three random fields (^*^P<0.05).

Next, we performed the transwell chamber invasion assays to evaluate the role of DcR3 in MFH cell invasion. In both cell lines, the number of invading cells in DcR3-siRNA transfected cells was decreased to 61% (TNMY1, Fig. 4A) and 64% (Nara-H, Fig. 4B) of that in control cells (^*^P<0.05). In addition, DcR3-Fc treatment after DcR3 knockdown significantly restored the invasiveness in both cell lines (^*^P<0.05, Fig. 4).

### DcR3 activated the PI3K/Akt pathway in MFH cells

**Figure 5:**
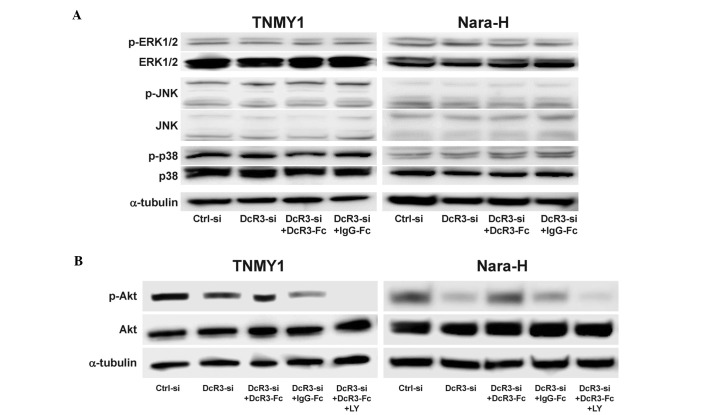
Effect of DcR3 on the activation of signaling kinases in MFH cells. (A) Immunoblot analyses were performed to detect the expression of ERK1/2, JNK, p38 and their phosphorylated forms in both MFH cell lines after siRNA transfection and recombinant Fc treatment. (B) Immunoblot analysis of Akt and phosphorylated Akt in both MFH cell lines treated with a PI3K inhibitor, LY294002 (LY) after siRNA transfection and recombinant Fc treatment.

Because DcR3 has been reported to be involved in the activation of various kinases (13–17), we evaluated the effect of DcR3 expression on the activation of signaling kinases in MFH cells. DcR3 knockdown and/or DcR3-Fc treatment did not affect the expressions of ERK1/2, JNK, p38 and their phosphorylated forms (Fig. 5A) and interestingly, DcR3 knockdown significantly decreased Akt phosphorylation in both MFH cell lines, which was restored by DcR3-Fc treatment (Fig. 5B). The expression of phosphorylated Akt was suppressed by DcR3-siRNA and/or pretreatment with the PI3K inhibitor, LY294002 (LY) (Fig. 5B).

### DcR3 regulated MMP-2 expression via the PI3K/Akt pathway in human MFH cells

**Figure 6:**
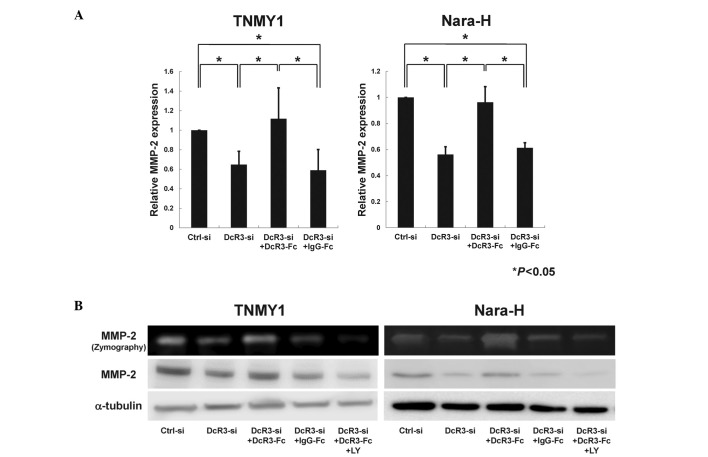
Effects of DcR3 on the expression and activation of MMP-2 in MFH cells. TNMY1 and Nara-H cells were transfected with either control or DcR3 siRNA and treated with recombinant Fc (DcR3 or IgG). DcR3-si+DcR3-Fc cells were also treated with the PI3K inhibitor, LY294002 (LY). (A) MMP-2 mRNA expression was analyzed by qRT-PCR (^*^P<0.05). (B) Immunoblot analysis and gelatin zymography for MMP-2.

As previous reports suggest that DcR3 may affect MMP-2 regulation via the PI3K/Akt pathway (12,17,39), we evaluated the effect of DcR3 inhibition on MMP-2 expression through the PI3K/Akt pathway in MFH cells. qRT-PCR analysis revealed that *MMP-2* mRNA expression was significantly suppressed by DcR3 knockdown and was restored by DcR3-Fc treatment in both MFH cell lines (^*^P<0.05, Fig. 6A). Immunoblot analysis and gelatin zymography showed that the protein expression and enzyme activity of MMP-2 were strongly reduced by siRNA knockdown of DcR3 (Fig. 6B). Consistent with the Akt phosphorylation (Fig. 5B), both the expression and enzyme activity of MMP-2 were increased by DcR3-Fc treatment and were significantly suppressed by LY294002 pretreatment (Fig. 6B).

## Discussion

DcR3 has been identified as a decoy receptor for FasL in lung and colon cancers (5) and recent studies have suggested that DcR3 acts as a modulator of cellular functions such as migration and invasion (10,11). It has been demonstrated that DcR3 overexpression is associated with tumor metastasis and prognosis of patients with various malignancies (5,18–26). These findings strongly indicate that DcR3 may be an attractive candidate as a novel therapeutic target for cancer treatment. We have previously reported the overexpression of DcR3 in musculoskeletal malignancies including MFH (21), however, the functional roles of DcR3 in MFH have not been studied. Therefore, we investigated the roles of DcR3 as a decoy receptor for FasL and as an effector molecule in migration and invasion in human MFH cells.

Because resistance to apoptosis is a characteristic property of malignant tumor cells to escape from immune attack by host immune systems (8), we first focused on the function of DcR3 as a decoy receptor for FasL. Many cancers, in spite of their Fas expression, can be resistant to FasL-induced apoptosis and several mechanisms may be responsible for the decreased sensitivity to FasL-induced apoptosis, including DcR3 (40). Yang *et al* reported that siRNA knockdown of DcR3 increased FasL-induced apoptosis in human pancreatic adenocarcinoma cells, which highly express DcR3 (38). In this study, we demonstrated that siRNA knockdown of DcR3 enhanced FasL-induced apoptotic activity in human MFH cell lines and that FasL treatment with DcR3 inhibition significantly suppressed MFH cell proliferation. In addition, DcR3-Fc treatment after DcR3 knockdown suppressed the increased FasL-induced apoptotic activity and induced MFH cell proliferation. The expression of Fas receptor in MFH cells was not affected by DcR3 knockdown or DcR3-Fc treatment. These results strongly suggest that DcR3 may contribute to MFH cell growth by inhibiting FasL-induced apoptosis as a decoy receptor.

Moreover, several studies have demonstrated that DcR3 functions as an effector molecule in various cells, independently of the FasL/Fas pathway, by regulating migration and invasion abilities (10,11), ERK stimulation in gastric cancers (41), increasing adhesion in monocytes via PI3K/Akt activation (17) and regulating proliferation and migration of HUVECs via MMP-2 regulation (12). These reports suggested that DcR3 has non-decoy functions, which are independent of the FasL/Fas apoptotic pathway. In the present study, we found that DcR3 inhibition significantly decreased cell migration and invasion in human MFH cells and that DcR3-Fc treatment significantly increased both abilities. These results indicate that DcR3 may regulate cell migration and invasion in human MFH.

Previous studies have also suggested the involvement of DcR3 in kinase phosphorylation (13–17). Therefore, we further investigated the effect of DcR3 expression on the activation of signaling kinases involved in migration and invasion in MFH cells. We revealed that Akt phosphorylation, which is inhibited by PI3K inhibition, was decreased by DcR3 knockdown and increased by DcR3-Fc treatment. Akt is a major signal transducer of the PI3K pathway, playing a pivotal role in the maintenance of cellular processes including cell growth, proliferation, survival and metabolism (42–44). An increase in Akt activity has been detected in various cancers (42–44). In addition, Akt signaling enhances MMP-2 activity and promotes cell migration and invasion (42). Therefore, we investigated the effect of DcR3 on MMP-2 activity in the PI3K/Akt pathway. Consistent with the Akt phosphorylation, DcR3 knockdown decreased MMP-2 expression and activity and DcR3-Fc treatment increased both the expression and activity of MMP-2, which were inhibited by PI3K inhibition. MMPs are thought to play a critical role in helping cancer cells invade through ECM degradation and form metastatic lesions (45). MMP-2, which is regulated by various kinases, is the most abundant among all MMPs (39,46) and has been reported to be upregulated in MFH (31,32). Activation of both PI3K/Akt pathway and MMP-2 are known to increase cell migration and invasion, leading to metastasis in various cancers (42,45). These findings indicate that DcR3 may regulate MMP-2 expression via activation of the PI3K/Akt pathway and that this regulation may be one of the important roles of DcR3 as an effector molecule facilitating the progression or metastasis of MFH.

**Figure 7:**
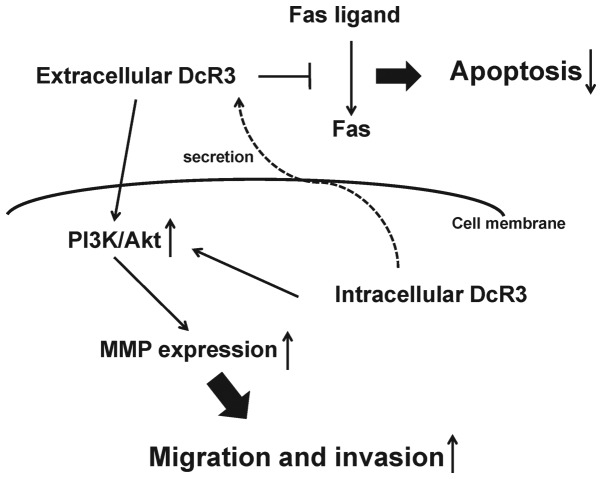
Schematic representation of the roles of DcR3 in MFH tumor progression. Decreased FasL-induced apoptotic activity by the decoy function of DcR3 contributes to tumor growth. The non-decoy function of DcR3 promotes cell migration and invasion by MMP-2 expression through the activation of the PI3K/Akt signaling pathway, resulting in accelerated tumor metastasis in MFH cells.

In conclusion, we demonstrated that in human MFH cells DcR3 may increase tumor progression as a decoy, promoting cell proliferation via inhibition of FasL-induced apoptosis and as a non-decoy, regulating cell migration and invasion by MMP-2 activation via the PI3K/Akt pathway (Fig. 7). Although further studies are needed to elucidate the roles of DcR3 in MFH tumor progression, our findings strongly indicate that DcR3 may be a potential therapeutic target in human MFH.

## Abbreviations

MFH: malignant fibrous histiocytoma
UPS: undifferentiated pleomorphic sarcoma
DcR3: decoy receptor 3
TNFR: tumor necrosis factor receptor
FasL: Fas ligand
LIGHT: lymphotoxin-like, exhibits inducible expression and competes with herpes simplex virus (HSV) glycoprotein D (gD) for HVEM, a receptor expressed by T lymphocytes
TL1A: TNF-like molecule 1A
PARP: poly (ADP-ribose) polymerase
HUVEC: human umbilical vein endothelial cell
MMP: matrix metalloproteinase
ERK: extracellular signal-regulated kinase
JNK: c-Jun N-terminal kinase
EBV: Epstein-Barr virus
HTLV-1: human T cell leukemia virus type 1
ECM: extracellular matrix
PI3K: phosphatidylinositol 3 kinase
